# Supplementary material for: Resolving the full spectrum of human genome variation using Linked-Reads
Source: Genome Res. 2019 Apr;29(4):635–45. doi: 10.1101/gr.234443.118 (PMC6442396; doi:10.1101/gr.234443.118)
Supplement: Supplemental Material [file supp_gr.234443.118_Supplemental_longranger-2.2.zip › longranger-2.2.tar.bz2!/lr2.2-public/lib/stan/cmdstan/stan/doc/api/html/agrad_2rev_2matrix_2log__determinant__ldlt_8hpp_source.html]

Stan: src/stan/agrad/rev/matrix/log\_determinant\_ldlt.hpp Source File


|  |  |
| --- | --- |
|  | Stan  2.5.0  probability, sampling & optimization |


- Main Page
- Related Pages
- Namespaces
- Classes
- Files

- File List
- File Members

All Classes Namespaces Files Functions Variables Typedefs Enumerations Enumerator Friends Macros Pages

- src
- stan
- agrad
- rev
- matrix

log\_determinant\_ldlt.hpp

Go to the documentation of this file.

1 #ifndef STAN\_\_AGRAD\_\_REV\_\_MATRIX\_\_LOG\_DETERMINANT\_LDLT\_HPP

2 #define STAN\_\_AGRAD\_\_REV\_\_MATRIX\_\_LOG\_DETERMINANT\_LDLT\_HPP

3

4 #include <stan/math/matrix/Eigen.hpp>

5 #include <stan/agrad/rev/var.hpp>

6 #include <stan/agrad/rev/matrix/LDLT\_alloc.hpp>

7 #include <stan/agrad/rev/matrix/LDLT\_factor.hpp>

8

9 namespace stan {

10  namespace agrad {

11  namespace {

12

22  template<int R,int C>

23  class log\_det\_ldlt\_vari : public vari {

24  public:

25  log\_det\_ldlt\_vari(const stan::math::LDLT\_factor<var,R,C> &A)

26  : vari(A.\_alloc->log\_abs\_det()), \_alloc\_ldlt(A.\_alloc)

27  { }

28

29  virtual void chain() {

30  Eigen::Matrix<double,R,C> invA;

31

32  // If we start computing Jacobians, this may be a bit inefficient

33  invA.setIdentity(\_alloc\_ldlt->N\_, \_alloc\_ldlt->N\_);

34  \_alloc\_ldlt->\_ldlt.solveInPlace(invA);

35

36  for (size\_t j = 0; j < \_alloc\_ldlt->N\_; j++) {

37  for (size\_t i = 0; i < \_alloc\_ldlt->N\_; i++) {

38  \_alloc\_ldlt->\_variA(i,j)->adj\_ += adj\_ \* invA(i,j);

39  }

40  }

41  }

42

43  const LDLT\_alloc<R,C> \*\_alloc\_ldlt;

44  };

45  }

46

47  template<int R, int C>

48  var log\_determinant\_ldlt(stan::math::LDLT\_factor<var,R,C> &A) {

49  return var(new log\_det\_ldlt\_vari<R,C>(A));

50  }

51  }

52 }

53 #endif

\_alloc

log\_determinant\_spd\_alloc< R, C > \* \_alloc

**Definition:** log\_determinant\_spd.hpp:30

LDLT\_alloc.hpp

stan::math::LDLT\_factor

**Definition:** LDLT\_factor.hpp:16

var.hpp

Eigen.hpp

stan::agrad::var

Independent (input) and dependent (output) variables for gradients.

**Definition:** var.hpp:27

stan::agrad::log\_determinant\_ldlt

var log\_determinant\_ldlt(stan::math::LDLT\_factor< var, R, C > &A)

**Definition:** log\_determinant\_ldlt.hpp:48

\_alloc\_ldlt

const LDLT\_alloc< R, C > \* \_alloc\_ldlt

**Definition:** log\_determinant\_ldlt.hpp:43

LDLT\_factor.hpp


---

[ Stan Home Page ]

*© 2011–2014,
Stan Development Team.*
